# Tribus: semi-automated discovery of cell identities and phenotypes from multiplexed imaging and proteomic data

**DOI:** 10.1093/bioinformatics/btaf082

**Published:** 2025-02-21

**Authors:** Ziqi Kang, Angela Szabo, Teodora Farago, Fernando Perez-Villatoro, Ada Junquera, Saundarya Shah, Inga-Maria Launonen, Ella Anttila, Julia Casado, Kevin Elias, Anni Virtanen, Ulla-Maija Haltia, Anniina Färkkilä

**Affiliations:** Research Program in Systems Oncology, University of Helsinki, Helsinki, 00290, Finland; Research Program in Systems Oncology, University of Helsinki, Helsinki, 00290, Finland; Research Program in Systems Oncology, University of Helsinki, Helsinki, 00290, Finland; Research Program in Systems Oncology, University of Helsinki, Helsinki, 00290, Finland; Research Program in Systems Oncology, University of Helsinki, Helsinki, 00290, Finland; Research Program in Systems Oncology, University of Helsinki, Helsinki, 00290, Finland; Research Program in Systems Oncology, University of Helsinki, Helsinki, 00290, Finland; Research Program in Systems Oncology, University of Helsinki, Helsinki, 00290, Finland; Research Program in Systems Oncology, University of Helsinki, Helsinki, 00290, Finland; Division of Gynecologic Oncology, Brigham and Women’s Hospital, Harvard Medical School, MA, Boston, 02115, United States; Dana-Farber Cancer Institute, Boston, MA, 02215, United States; Research Program in Systems Oncology, University of Helsinki, Helsinki, 00290, Finland; Department of Pathology, University of Helsinki and HUS Diagnostic Center, Helsinki University Hospital Helsinki, 00290, Finland; Research Program in Systems Oncology, University of Helsinki, Helsinki, 00290, Finland; Department of Obstetrics and Gynecology, Helsinki University Hospital, Helsinki, 00290, Finland; Research Program in Systems Oncology, University of Helsinki, Helsinki, 00290, Finland; Department of Obstetrics and Gynecology, Helsinki University Hospital, Helsinki, 00290, Finland; Institute for Molecular Medicine Finland, Helsinki Institute of Life Sciences, University of Helsinki, Helsinki, 00290, Finland

## Abstract

**Motivation:**

Multiplexed imaging and single-cell analysis are increasingly applied to investigate the tissue spatial ecosystems in cancer and other complex diseases. Accurate single-cell phenotyping based on marker combinations is a critical but challenging task due to (i) low reproducibility across experiments with manual thresholding, and, (ii) labor-intensive ground-truth expert annotation required for learning-based methods.

**Results:**

We developed Tribus, an interactive knowledge-based classifier for multiplexed images and proteomic datasets that avoids hard-set thresholds and manual labeling. We demonstrated that Tribus recovers fine-grained cell types, matching the gold standard annotations by human experts. Additionally, Tribus can target ambiguous populations and discover phenotypically distinct cell subtypes. Through benchmarking against three similar methods in four public datasets with ground truth labels, we show that Tribus outperforms other methods in accuracy and computational efficiency, reducing runtime by an order of magnitude. Finally, we demonstrate the performance of Tribus in rapid and precise cell phenotyping with two large in-house whole-slide imaging datasets.

**Availability and implementation:**

Tribus is available at https://github.com/farkkilab/tribus as an open-source Python package.

## 1 Introduction

Multiplexed imaging techniques at single-cell resolution, such as tissue-based cyclic immunofluorescence (t-CyCIF) (Lin *et al.* 2018), co-detection by indexing (CODEX) (Black *et al.* 2021), and multiplexed ion beam imaging by time of flight (MIBI-TOF) (Keren *et al.* 2019), offer significant advantages for studying tissue architecture. These techniques enable researchers to measure dozens of proteins at single-cell resolution while preserving spatial origin information in tissue sections, providing novel insights into cellular phenotypes and tissue behaviors (Spitzer and Nolan 2016). Multiplexed images require a sequence of processes to extract single-cell measurements, including image registration, stitching, cell segmentation, and quantification (Schapiro *et al.* 2022). Cell phenotyping is typically the final step before downstream analyses and often serves as the bottleneck in realizing the full potential of multiplexed images. The main challenges in cell-type phenotyping from multiplexed images include reproducibility limitations and unexpected marker combinations.

Classic methods for cell phenotyping such as manual gating and clustering, are reproducibility limited. Gating (Staats *et al.* 2019) requires visualizing and manually setting marker expression thresholds for each marker in each sample. These hard thresholds are experiment-specific and cannot be reused across different batches. As the marker panel sizes and sample numbers increase, gating becomes time-consuming and unfeasible (Verschoor *et al.* 2015). Clustering algorithms, such as PhenoGraph (Levine *et al.* 2015), Leiden (Traag *et al.* 2019), and DBscan (Ester *et al.* 1996), have been applied for automatic data exploration (Liu *et al.* 2019). However, manual verification is still necessary to assign meaningful cell types to the resulting clusters. To achieve deeper profiling of cell types, over-clustering and subsequent merging of clusters is often required, a process that is both computationally intensive and time-consuming.

Several automated cell-type annotation approaches have been developed to overcome the reproducibility limits. For example, CellSighter is a supervised deep convolutional neural network-based algorithm for automatic cell phenotyping which requires expert-labeled images for training (Amitay *et al.* 2023). Another similar solution, MAPS is a supervised deep learning-based method that is computationally lighter than CellSighter (Shaban *et al.* 2024). These methods require pre-training on manually labeled datasets; however, the measured protein combinations (marker panel design) are experiment-specific, making it difficult to generate general reference populations for each cell type. In cases of unexpected marker combinations, the algorithms are required to be retrained in different scenarios.

To address the above challenges, we introduce a novel cell-type caller named Tribus, which incorporates the widely used self-organizing map (SOM) (Kohonen 1982) unsupervised clustering method with a unique scoring function to assign cell types according to prior biological knowledge. Tribus requires only a cell measurement matrix and a prior knowledge table as inputs, without the need for training on expert annotations, and enables reproducible, automatic cell phenotyping across various multiplexed imaging and proteomic datasets. Tribus enables users to easily conduct analyses, visualize results, and perform quality control through an integrated Napari widget. We validate Tribus’s accuracy on four public multiplexed imaging and suspension mass cytometry datasets. We then compare Tribus’s performance to three other similar prior knowledge-based cell-type identification approaches: ACDC (Lee *et al.* 2017), Astir (Geuenich *et al.* 2021), and Scyan (Blampey *et al.* 2023), and demonstrate its utility in analyzing two large in-house t-CyCIF datasets. Tribus represents a novel user-friendly framework for semi-automated cell-type calling in multiplexed imaging and proteomics datasets.

## 2 Materials and methods

### 2.1 Overview of Tribus

**Figure 1.**
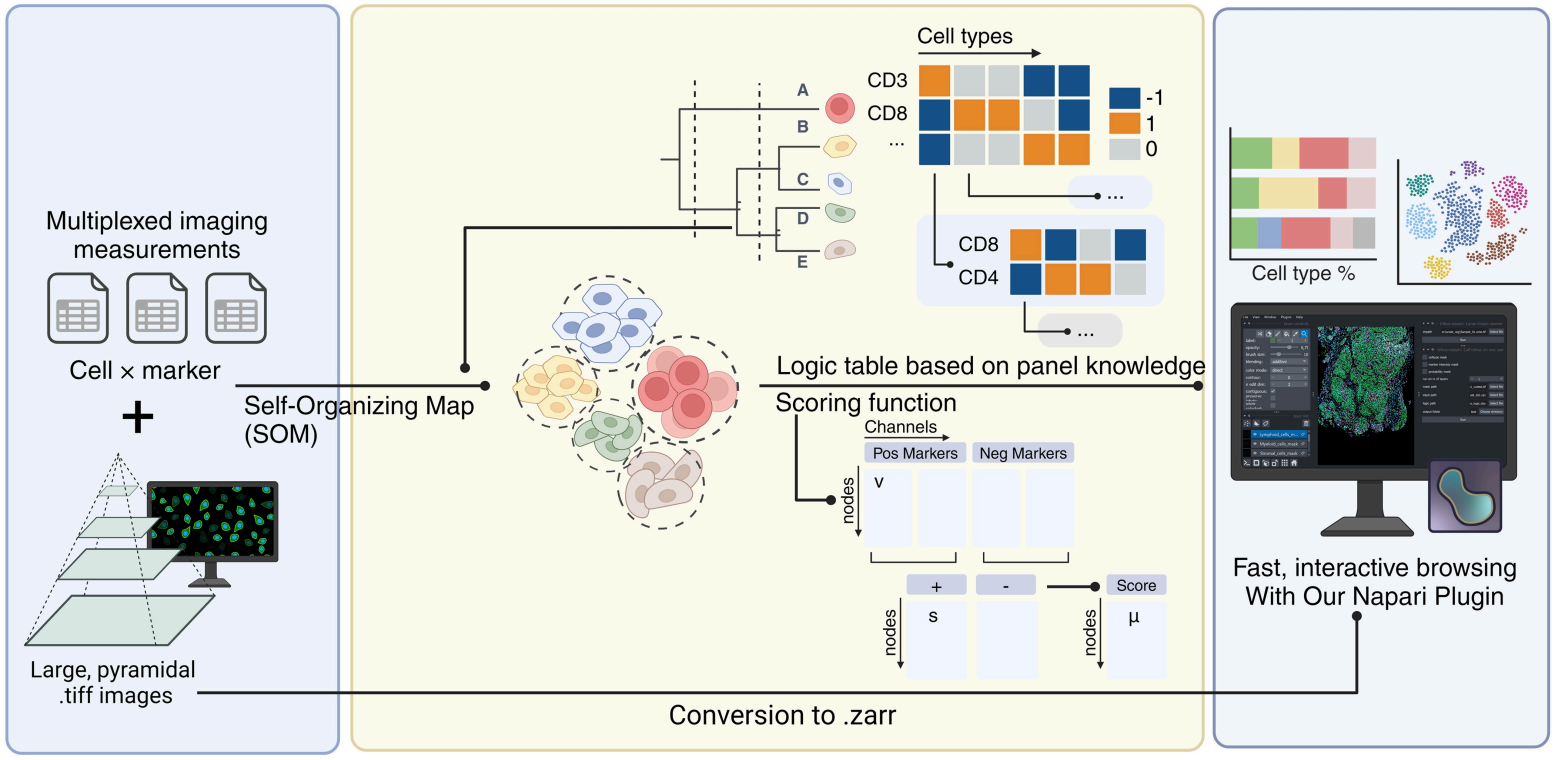
Overview of Tribus architecture. Tribus processes multiplexed imaging data by using a SOM to cluster cells and a logic table based on panel knowledge to score and classify cell phenotypes. Results can be visualized with a Napari plugin for interactive exploration and quality control.

Tribus is a hierarchical framework for cell-type assignment in multiplexed image datasets based on prior panel knowledge (Fig. 1). It requires two inputs: a single-cell marker expression table and a logic table of cell phenotypes based on prior knowledge. The marker expression table is a matrix where rows represent individual cells and columns correspond to measured features, such as marker expressions. The logic table is another matrix $L\left(c_{i},m_{j}\right)$ containing values $\left[-1,0,1\right]$, where $c_{i}$ denotes the $i_{th}$ cell type and $m_{j}$ the $j_{th}$ marker. A value of 1 indicates a marker is expected to be present in a certain cell type, −1 indicates absence and 0 represents neutral or unknown markers. Each cell type must have at least one positive marker in the logic table (Supplementary Tables S1–S3). The output of Tribus includes annotated cell types for each cell, which can be used to evaluate annotation quality or perform downstream analysis (Supplementary Table S4). The original image is included in “human-in-the-loop” phase for quality control.

### 2.2 Unsupervised clustering in Tribus

Tribus uses an unsupervised SOM method for clustering based on the MiniSOM package (Vettigli 2023). SOM can represent a high-dimensional input space as a map consisting of components called “nodes.” Quantization error (Q) was used to evaluate the algorithm performance, calculated by determining the average distance of the sample vectors ($\vec{x}$) to the cluster centroids ($\vec{w}$).


$$Q=\;\frac{1}{m}\sum_{q=1}^{m}{\left\|{\vec{x}}^{q}-{{\vec{w}}_{i}}^{q}\right\|}^{2}$$

Quantization errors can only be compared under the same grid size (Pölzlbauer n.d.). To determine the optimal grid size, we used the approach of Vesanto (Vesanto and Alhoniemi 2000): $G=5\sqrt{N}$, where N is the input data size (i.e. number of cells). The parameters of the SOM include σ (the spread of the neighborhood function) and the learning rate. Those parameters can be set by users or optimized by minimizing the quantization error with the hyperparameter tuning module based on the package hyperopt (Bergstra *et al.* 2013), where the objective function was to minimize the quantization error, and the search space for σ and the learning rate ranged from 0.001 to 5.

During analysis, Tribus first generates clusters/grids from the input data and then assigns each cluster to a certain cell type based on the logic table. Cell type assignment is performed hierarchically, meaning Tribus first assigns lower-level cell types followed by higher-level cell subtypes to create more precise categories. If the number of cells in the subset exceeds the user-defined threshold, Tribus will still generate clusters. Otherwise, Tribus directly calculates the scoring function for each cell type at the single-cell level.

### 2.3 Cell type assignment by scoring functions

After SOM clustering, a node matrix $N\left(n_{k},v_{j}\right)$ is generated, where $v_{j}$ is calculated as the median expression of the marker $m_{j}$ in cluster $n_{k}$. We designed a scoring function based on the squared error concept, similar to the QueryStarPlot function of the FlowSOM package (Van Gassen *et al.* 2015). This function calculates the score $s_{i}(n_{k})$ of a certain cell type i for each node k.


$$s_{i}\left(n_{k}\right)=\;\frac{1}{k}\sum_{j}^{\;}\;\left[1-{\left(v_{ij}-\hat{v_{ij}}\right)}^{2^{\prime }}\right]$$

Here, $\hat{v_{ij}}$ is determined based on the prior knowledge logic table $L(c_{i},\;m_{j})$ as follows.


vij^=P99vij, L(ci, mj)=1min⁡(vij), L(ci, mj)=-1

In this equation, $P_{99}\left(v_{ij}\right)\;$represents the 99th percentile of the $v_{j}$ for cell type i, chosen instead of the maximum value to improve robustness against outliers. The logic table $L(c_{i},\;m_{j})$ indicates the relationship between cell type $c_{i}$ and marker $m_{j}$. The cell type $c_{i}$ assigned to node $n_{k}$ is the one that maximizes the score, determined as $c_{i}=\mathrm{argma}x_{s_{i}}f\left(n_{k},s_{i}\right)$. Note that the cell-type assignment process in Tribus accounts for ambiguous results. If the maximum score of a cluster falls below a predefined threshold, the cluster is labeled as the “other” cell type. Similarly, if the difference between the maximum and second maximum score is smaller than a predefined threshold, the cluster will be labeled as “undefined.”

### 2.4 Napari plug-in

To efficiently evaluate cell-type labeling results, we developed a custom plugin integrated with the Napari (Ahlers *et al.* 2023) framework. This plugin enables users to run Tribus on one sample at a time, display results simultaneously, or load previously saved data. We incorporated the ZARR format to overcome computational limitations associated with large datasets.

The key functionalities of the Napari plugin include:

1. Cell-type mask visualization: The plugin sorts and displays different cell-type labels as separate layers using visually distinct colors, allowing the user to overlay them with imaging data for quality control. This function is also available in a stand-alone Jupyter Notebook.
2. Probability score visualization: cell masks are represented as a color gradient of the probability score assigned by the algorithm. This allows the user to identify and review ambiguous cells and assess the assigned “other” and “undefined” thresholds.
3. Marker intensity visualization: The median expression levels of the selected markers are represented through gradient shading on the segmentation mask, enabling users to visually assess the results and identify potential biases.

### 2.5 Methods for comparison and evaluation metrics

We compared the performance of Tribus with three similar prior knowledge-based cell-type calling tools: ACDC, Astir, and Scyan. We evaluated overall cell type annotation performance by comparing the Rand Index (Rand 1971), weighted F1 score (Powers 2020), accuracy, and Cohen’s kappa coefficient. We also compared the Matthews correlation coefficient (MCC), given the size variability of some cell types. All the above metrics were calculated using functions provided by Scikit-learn (Pedregosa *et al.* 2011).

### 2.6 Benchmarking datasets

#### 2.6.1 Public datasets

We chose four public suspension mass cytometry and multiplexed imaging datasets with ground truth labels to evaluate the performance of Tribus (Table 1). The AML dataset contains single-cell proteomic profiles of human bone marrow from patients with acute myeloid leukemia (AML) and healthy adult donors (Levine *et al.* 2015). The “NotDebrisSinglets” cell type was excluded from the analysis. The BMMC dataset was derived from bone marrow mononuclear cells (BMMCs) (Bendall *et al.* 2011). According to the research, Erythroblasts, megakaryocyte platelets, and myelocytes were merged as an unknown population and removed from the analysis. All “NotGated” cells were excluded (*N* = 61 725 cells). The ductal carcinoma in situ (DCIS) dataset containing 79 clinically annotated surgical resections (Risom *et al.* 2022), including normal breast tissue (*N* = 9, reduction mammoplasty), primary DCIS (*N* = 58), and invasive breast cancer (IBC) (*N* = 12). Cell types were merged into endothelial, epithelial, fibroblast, immune, and myoepithelial during low-plex cell phenotyping. HuBMAP is a published co-detection by indexing (CODEX) imaging dataset (Hickey *et al.* 2023). Only donor 004 was manually annotated and, therefore, used in this study. For low-plex cell phenotyping, cell types were merged into epithelial, stromal, lymphoid, and myeloid. All public datasets were already pre-processed. Suspension mass cytometry datasets were normalized by dividing the maximum intensity values determined as the 99.5th percentile of cells. Multiplexed imaging datasets were normalized with log and z-score normalization, 99.9th percentile outlier removal, and co-factor 5 arcsinh transformation (only in DCIS dataset).

**Table 1.** Summary of the public benchmarking datasets.

Data	Methods	No. of marker	No. of cell type	No. of cell	No. of tissue
AML	Suspension mass cytometry	32	14	104 184	Human bone marrow
BMMC	Suspension mass cytometry	13	24	61 725	Human bone marrow
DCIS	MIBI-TOF	37	23	69 672	Primary ductal carcinoma in situ (DCIS) and normal human breast tissue
HubMAP	CODEX	54	25	110 635 (colon), 137 652 (small bowel)	Human healthy intestine (colon and small bowel)
NACT	t-CyCIF	36	8	976 082	Two tumor sections after neoadjuvant chemotherapy (NACT) and one treatment-naive biopsy
Oncosys-Ova	t-CyCIF	14	6	∼10.5 M	Homologous recombination-proficient HGSC samples

#### 2.6.2 In-house datasets

We generated two in-house high-grade serous ovarian cancer (HGSC) datasets using t-CyCIF. The NACT dataset contains three images: two tumor sections after neoadjuvant chemotherapy (NACT) and one treatment-naive biopsy. The NACT dataset was stained using a 36-plex antibody panel (Supplementary Table S6). The Oncosys-Ova dataset contains 21 HGSC samples stained with a 14-plex panel (Supplementary Table S7). Marker expression tables were preprocessed using log transformation, *z*-score normalization, 99.9 percentile outlier removal, and co-factor 5 arcsinh transformation (Hickey *et al.* 2021) before phenotype assignment. In the NACT dataset, 976 082 cells were annotated using a cell phenotyping logic table based on the panel design. For the Oncosys-Ova dataset, approximately 10.5M cells were annotated. Following the ethical standards from the 1975 Declaration of Helsinki, every patient from ONCOSYS-Ova trial (NCT06117384) provided informed written consent to the collection, storage, and analysis of the samples and subsequent data. For the NACT dataset, the Mass General Brigham Institutional Review Board approved using human tissue samples. Informed consent was waived due to the use of archival samples and anonymization of the material.

## 3 Results

### 3.1 Tribus recovers fine-grained cell types as accurately as human experts

**Figure 2.**
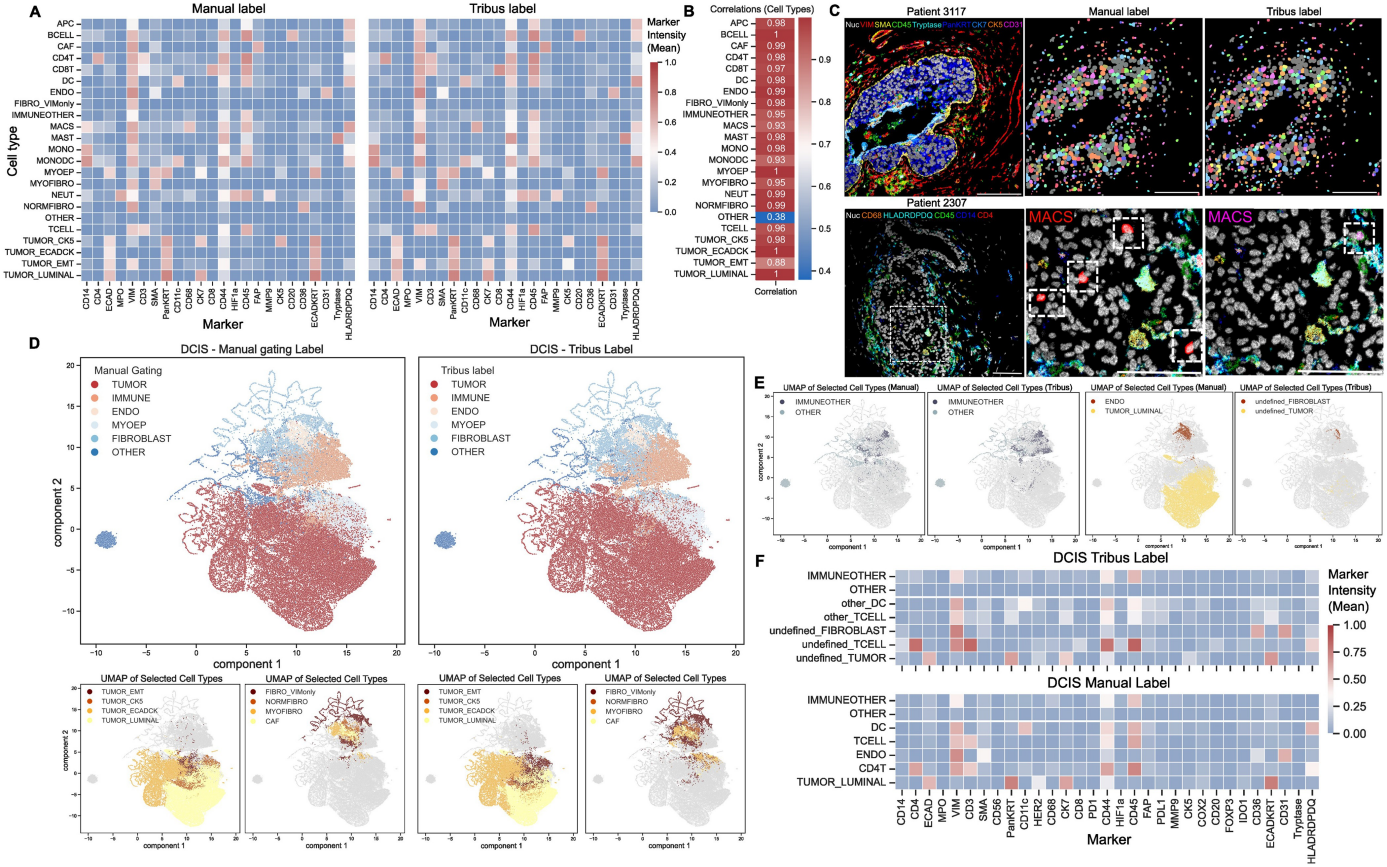
Tribus applied on the public DCIS dataset. (A) Heatmaps showing the mean marker intensity of manually gated cell populations and Tribus-classified populations from the DCIS dataset are similar. (B) Heatmap showing high Pearson correlation scores among cell types between ground truth labels and Tribus labels. (C) Representative MIBI images. The upper image is from patient 3117 (DCIS tumor) with a nine-color overlay of markers related to major cell types. Cell-type masks from the manual label and Tribus show few differences. The lower image is from patient 2307 (Normal tissue) with a six-color overlay of MACS-related markers. Compared to the MACS cell masks of manual and Tribus, some MACS cells from manual labels do not have related marker expression, which was corrected in Tribus annotation (canonical marker combinations were observed). (D) UMAP representation of manual and Tribus labels on the DCIS dataset. Cell types were color-coded based on original and Tribus annotation, the same cell type label was assigned the same color. (E) Comparing (1) undefined and other cell populations with ground truth labels, (2) undefined cellular subpopulations from Tribus annotation with relevant manual labeled cell populations. (F) Heatmaps comparing the mean marker intensity of manually gated cell populations and the ambiguous cell populations from Tribus annotation. Tribus can identify the same ambiguous populations and discover phenotypically new subtypes.

To evaluate Tribus’s ability to recover canonical cellular populations, we applied it to the benchmark DCIS dataset. Tribus successfully identified all populations highlighted in the study (Supplementary Fig. S2A). The mean marker intensities of Tribus-labeled cellular populations matched those of human-labeled populations (Fig. 2A), with high pair-wise Pearson correlation scores exceeding 0.95 across the cell types (Fig. 2B). One notable difference was observed in the MACS (macrophages) population, where Tribus labels displayed a lower median intensity of CD14 compared to manual gating. This discrepancy may be due to the fact that CD14 was not used for MACS identification in DCIS research, and thus CD14 was not constrained to be positive for MACS cells in the logic table. We mapped cell masks back to the original tiff images with a nine-color overlay of cell identity-related markers. The cell masks were consistent across all major cell types in Tribus labels and manual labels. False positive MACS in manual labels were corrected by Tribus (Fig. 2C). UMAP visualization of all cell types from DCIS datasets showed that Tribus accurately annotated the majority of cells (Fig. 2D). Using the manual labels as ground truth, Tribus achieved precision scores between 0.7 and 0.8, average recalls around 0.6, and F1 scores between 0.6 and 0.7 across most cell types (Supplementary Fig. S1B). These results indicate that Tribus can recover and annotate fine-grained cell types with a level of accuracy comparable to human experts.

### 3.2 Tribus can target ambiguous populations and discover phenotypically new subtypes

One challenge for prior knowledge-based cell-type calling methods is discovering ambiguous categories that are difficult to predefine in the logic table. For example, a group of unknown cells with low intensity across all markers. The DCIS dataset provides a good example for exploring ambiguous populations, as it includes the “IMMUNEOTHER” and “OTHER” cell types in the manual gating labels. The “OTHER” cell type has low intensities across all markers, while “IMMUNEOTHER” lacks specific immune subtype marker expression.

We verified that Tribus can successfully target ambiguous populations by adjusting the decision thresholds in the scoring function. We explored threshold settings and found Tribus is robust to different threshold values within a certain range (Supplementary Fig. S3A and B). We selected an undefined_threshold of 0.001 and other_threshold of 0.04 for the analysis of the DCIS dataset. From the UMAP visualization, we observed that the same “IMMUNEOTHER” and “OTHER” clusters retained the same local structures (Fig. 2E). The marker expression heatmap demonstrates that Tribus-targeted ambiguous cell types share the same marker expression profiles as manually gated cell types (Fig. 2F). We also discovered new clusters, including undefined tumors, fibroblasts, T-cells, and DCs (Fig. 2D, Supplementary Fig. S2C). We found phenotypically new clusters of DCs, T-cells, and CD4 T cells using the mean marker expression heatmap (Fig. 2F). We observed novel marker expression combinations and cellular subtypes were validated by mapping cell masks back to the original images. We successfully identified CD36+CD31+ fibroblasts, HER2-luminal subtypes, and undefined T-cells which exhibit higher phenotypic marker expression than typical CD4 T-cells (Supplementary Fig. S4A–C). These findings demonstrate that Tribus is not only effective in identifying ambiguous cell populations but also capable of discovering phenotypically novel cell subtypes. We suggest that Tribus could serve as a starting point for uncovering novel cell states.

### 3.3 Tribus outperforms other similar methods

We benchmarked the performance of Tribus against three other approaches: ACDC, Scyan, and Astir. We chose these tools for benchmarking because they are all prior knowledge-based cell phenotyping methods, each designed for specific high-dimensional data types.

To ensure a fair comparison, we minimized experimental differences by using consistent prior knowledge tables and avoiding biased parameter settings. For AML and BMMC datasets, we used the knowledge tables provided by ACDC and Scyan, which were reformatted into the logic tables/YAML files accordingly for Tribus/Astir. For the DCIS and HubMAP datasets, where no predefined knowledge tables were available, we generated tables for all methods based on the panel information provided in the original studies. Analysis parameters for each method were carefully selected to align with published recommendations or example scripts. The ACDC analyses were performed with the parameters (n_neighbor = 10, thres = 0.5) from the example scripts. The parameters for the Astir analysis were chosen (max_epochs = 1000, learning_rate = 2e-3, initial_epochs = 3) based on the Colab tutorial provided in the original study. The parameters for Scyan analysis were chosen based on the tutorial provided in the GitHub repository.

**Figure 3.**
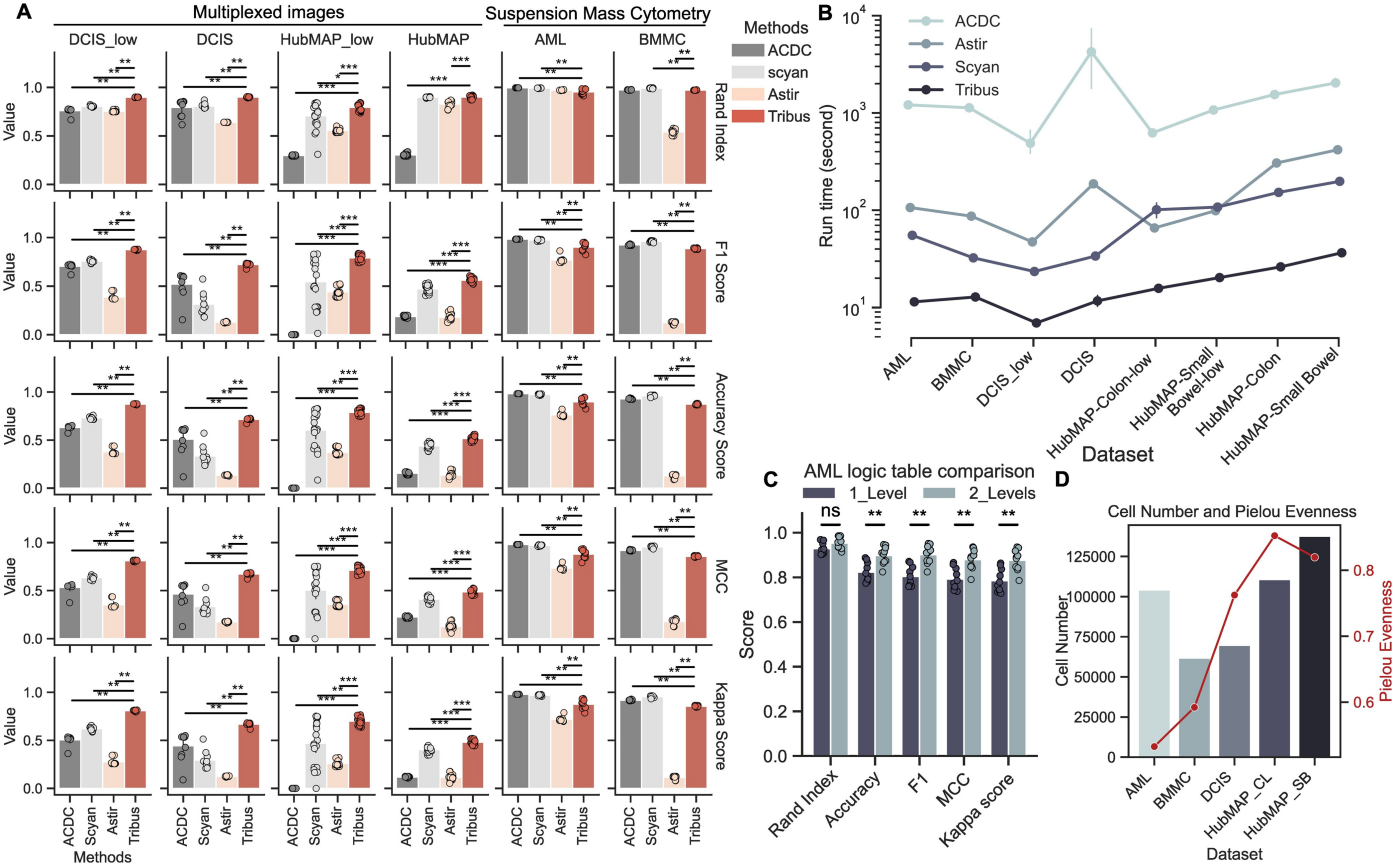
(A) Performance comparison of Tribus and other similar methods on four datasets (AML, BMMC, DCIS-lowplex, DCIS, HubMAP-lowplex, HubMAP) using five metrics for each. All analyses were repeated ten times. Using standard deviation for the error bar. Two-sided Wilcoxon signed-rank test was used for statistical testing for the differences among metrics of Tribus and other benchmarking results. ns: not shown. *: *P* < .05, **: *P* < .01, ***: *P* < .001. (B) Models running time comparison. All analyses were repeated ten times and used standard deviation as the error bar. (C) Compare Tribus performance under different logic tables for the AML dataset, using standard deviation as the error bar. Each analysis was repeated ten times. Two-sided Wilcoxon signed-rank test was used for statistical testing for the differences among metrics of Tribus performance with different logic tables. ns: not shown. *: *P* < .05, **: *P* < .01, ***: *P* < .001. (D) Data complexity comparison over public benchmarking datasets, showing the number of cells and Pielou’s evenness index. Higher Pielou’s evenness index represents high diversity and high evenness of cell populations.

Benchmarking experiments showed that Tribus outperformed the other methods in terms of efficiency and accuracy. Tribus outperforms Astir, ACDC, and Scyan methods across metrics on highly multiplexed imaging datasets, DCIS and HubMAP, in both high- and low-plex cell phenotyping. In suspension mass cytometry datasets such as AML and BMMC, most metrics were however statistically lower compared to compared to ACDC and Scyan, which were designed specifically for cell phenotyping on mass cytometry datasets (Fig. 3A, Supplementary Table S5). Importantly, Tribus was significantly faster, with runtimes an order of magnitude shorter than other methods (Fig. 3B). Tribus successfully identified all cell types highlighted in the four public datasets (Supplementary Figs S5 and S6).

We then explored how the structure of the logic table influenced performance. Using the AML dataset as an example, we applied Tribus analysis with (i) a logic table with only one global level and (ii) a logic table that includes major cell types at the global level and sub-phenotypes (e.g. CD16− and CD16+ NK cells) at the second level (Supplementary Table S1–S3). We repeated the Tribus analysis ten times, calculated the average metrics, and visualized the results for each logic table configuration. When adjusting the logic table for the AML dataset, using a hierarchical logic table improved accuracy and increased the F1 score by 0.1 (Fig. 3C).

Finally, we calculated Pielou’s evenness index to illustrate the increasing complexity across benchmarking datasets (Fig. 3D). Tribus’s performance remained relatively good and stable from suspended single-cell bone marrow datasets to highly complex human intestine slide datasets. In summary, Tribus outperformed other similar methods in both accuracy and efficiency, particularly in highly multiplexed imaging datasets, while maintaining robust results across different cell phenotyping contexts.

### 3.4 Tribus yields rapid and accurate cell phenotyping in large whole-slide image datasets

We evaluated the performance of Tribus on large in-house multiplexed image datasets. For the Oncosys-Ova dataset, we used a one-level logic table consistently across all 21 samples (Supplementary Fig. S7A). We applied a four-level logic table to the NACT dataset (Supplementary Fig. S7B).

**Figure 4.**
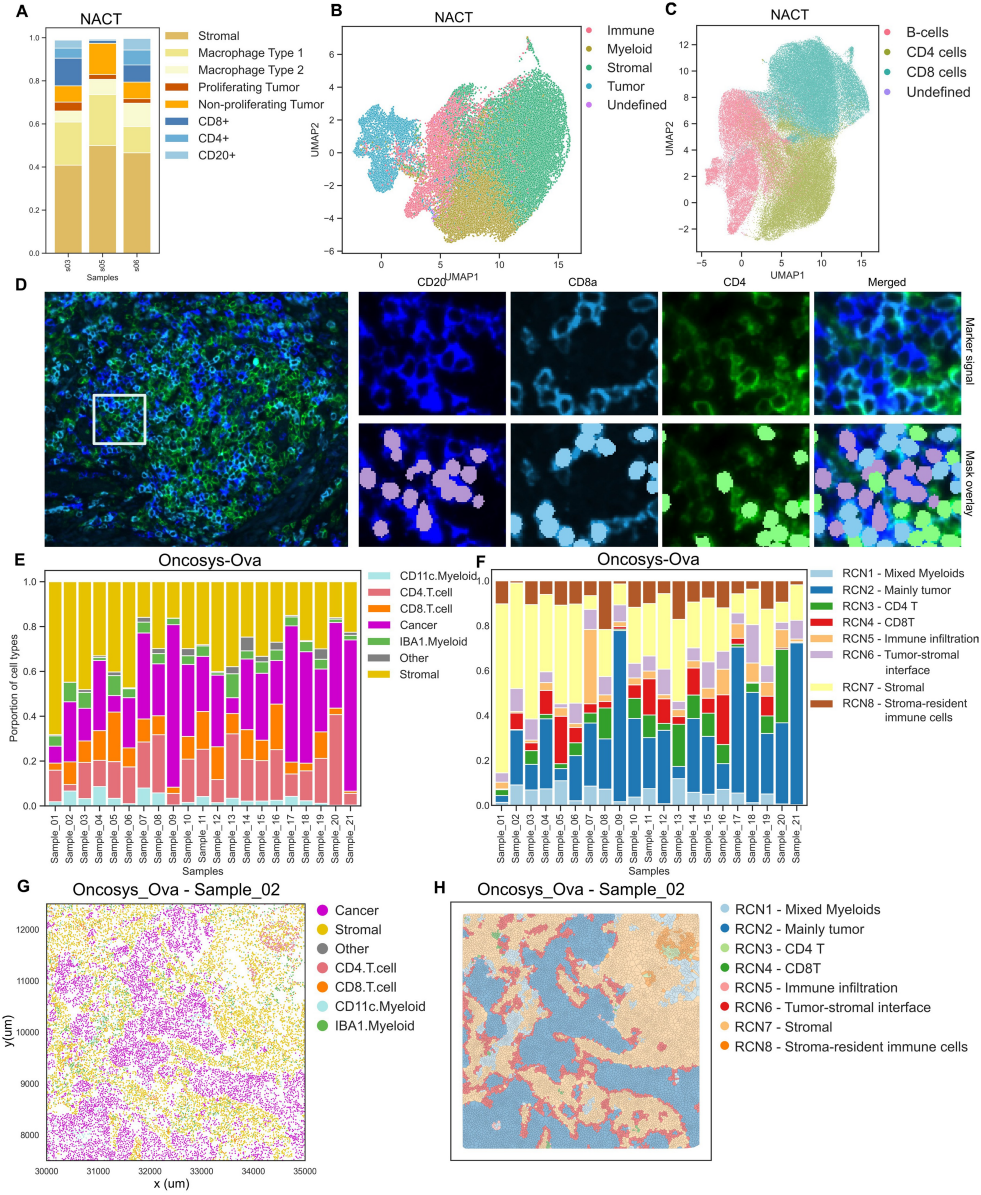
(A) Annotated barplot of the cell phenotype compositions per sample in the NACT dataset. (B) UMAP visualizes distinct cell populations colored by the major cell types in the NACT dataset. (C) UMAP visualizes distinct cell populations colored by the immune subtypes in the NACT dataset. (D) Representative tCyCIF image (sample 06) of the NACT dataset, showing the nuclei and representative tumor and immune markers. Tribus accurately identified the CD20+, CD8a+, and CD4+ populations within a Tertiary Lymphoid Structure (TLS) despite the dense organization of these sub-phenotypes. (E) The stacked barplot shows cellular proportion in all samples. (F) Stacked barplot shows various RCN proportions in samples. (G) The scatter plots show the local tissue architecture colored by cell types. (H) The Voronoi plot visualizes the structures of RCNs in the corresponding region of figure (G).

In the NACT dataset, Tribus successfully identified major cell phenotypes and subtypes, which we characterized based on the available marker panel (Fig. 4A). The UMAP projection showed distinct cell-type populations in the NACT dataset (Fig. 4B and C) and minimal batch effects in phenotyping analysis (Supplementary Fig. S7C). The multiplexed t-CyCIF image from sample 06 of the NACT dataset displayed nuclei and representative tumor and immune markers. Tribus accurately identified the CD20+, CD8a+, and CD4+ cell populations within a Tertiary Lymphoid Structure (TLS) despite the dense organization of these sub-phenotypes (Fig. 4D). Tribus also annotated the proliferating subpopulation of tumor cells and tumor-infiltrating CD8+ cells from a dense area (Supplementary Fig. S7D), indicating that Tribus can generate accurate phenotype labels in complex tissue architectures.

In the Oncosys-Ova dataset, Tribus accurately identified six cell types with substantial cellular proportion heterogeneity among samples (Fig. 4E). UMAP projections showed separated cell populations and low batch effects (Supplementary Fig. S8A). The heatmap showed canonical marker expression combinations for each identified cellular population (Supplementary Fig. S8B). We used Scimap (Nirmal and Sorger 2024) to calculate the fractions of neighboring cell types within a radius of 100 μm, then applied k-means clustering (*k* = 10) on the neighborhood matrix and generated eight Recurrent cellular neighborhoods (RCN) (Fig. 4F). The RCNs are distinct spatial domains within the tissue and successfully capture relevant spatial biology based on Tribus cell phenotypes (Supplementary Fig. S8C). The representative presentation of the RCNs across tissue uncovered the complex tissue architecture such as the tumor-stromal interface and tumor-infiltrating lymphocytes cells (Supplementary Fig. S8D). Tribus-based spatial analysis enabled us to map the tumor-stromal interface in complex tumor-rich regions (Fig. 4G, Supplementary Fig. S8D) and plot the stroma-resident immune cells in a stromal-rich region (Supplementary Fig. S8E). These results suggested that Tribus adapts well in the workflow of cell phenotyping on large whole-slide images and downstream spatial pattern analysis.

## 4 Discussion

Cell-type calling is a crucial step in high-dimensional image analysis. The growing complexity and increasing number of panels in high-dimensional data necessitate the development of reproducible and automated cell-type calling approaches. In this study, we developed Tribus, a semi-supervised cell-type calling analysis framework for multiplexed imaging datasets. Tribus offers advantages in efficiency, accuracy, user-friendliness, and reproducibility without the need for training using manual labels.

Tribus was designed as a semi-automated “human-in-the-loop” cell-type caller for multiplexed imaging data, incorporating biological knowledge from the panel design into the analysis. This was achieved through carefully designed scoring functions based on marker expression per grid, generated by unsupervised clustering to minimize bias. When the number of cells in the clusters was below the user-defined threshold, Tribus skipped generating the clusters and calculated scores at the single-cell level. This flexible scoring function calculation strategy enabled the discovery of rare cell types. Tribus was integrated with Napari, and a plugin was provided to enable one-click import of the cell-type identification results, significantly enhancing the simplicity of interactive quality control. This integration allows for convenient operation by users who are unfamiliar with programming.

Tribus demonstrated robust performance across diverse datasets, particularly excelling in multiplexed imaging datasets, where it outperformed other methods. However, on suspension mass cytometry datasets such as AML and BMMC, certain metrics were statistically lower compared to some of the methods specifically designed for this data type. For instance, AML ground truth labels were generated using Phenograph, a clustering-based method that inherently favors approaches like ACDC, which follows a similar clustering-based strategy. In contrast, Tribus uses a knowledge-based classification approach, offering greater generalizability, interpretability, and stability across various datasets while maintaining efficiency. However, it should be acknowledged that Tribus exhibits greater stability across diverse datasets, as evidenced by its consistent performance metrics. Additionally, Tribus is less time-consuming, which is advantageous for scalability and practical application in large datasets.

Cell-type annotation from bioimages presents inherent challenges due to imperfect cell segmentation and collateral spillover. Expanding nuclei masks by a few pixels can enhance cytoplasmic marker visibility, as it allows better signal capture when cells express these markers. However, in dense tissue areas, this might increase spillover, highlighting the importance of the hierarchically structured logic table. Typically, mild spillover affects only part of a cell, whereas a true signal produces a more uniform expression pattern and a higher mean fluorescence intensity. Tribus was designed to account for both positive and negative components of the expected marker expression, and it also includes the option to set markers with expected false-positive expressions as neutral. Thus, careful design of the logic table and its hierarchy in Tribus can aid in cell phenotypic separation, as only a subset of cells is considered at the lower hierarchy levels during phenotype assignment.

However, Tribus is not without limitations. The performance of Tribus is strongly tied to the quality of the input dataset and the prior knowledge of expected cell types in the user-defined initial logic table. To assign a uniform logic table, the samples should have even staining patterns both within and across slides. Uneven staining patterns and antibodies with suboptimal signal-to-noise ratios can significantly affect the results. For such suboptimal datasets, users can create a hierarchical logic table where major cell phenotypes with clear marker signals are separated at higher levels. This can result in more accurate labeling if distinct areas of the image are affected. Additionally, Tribus cannot identify cell types not included in the input logic table, but it can return undefined- or other-cell types for further exploration.

Overall, we propose Tribus as a fast, accurate, and user-friendly cell-type identification method that can be integrated into multiplexed image analysis frameworks.

## Supplementary Material

btaf082_Supplementary_Data

## Data and availability

The Tribus package is publicly available on Github: https://github.com/farkkilab/tribus. The code of Tribus was archived in Zenodo: https://doi.org/10.5281/zenodo.14725051. The datasets and scripts used to repeat the results are publicly available at Synapse: https://www.synapse.org/Synapse: syn53754523/files/.
