# Supplementary material for: Tribus: semi-automated discovery of cell identities and phenotypes from multiplexed imaging and proteomic data
Source: Bioinformatics. 2025 Feb 21;41(3):btaf082. doi: 10.1093/bioinformatics/btaf082 (PMC11932726; doi:10.1093/bioinformatics/btaf082)
Supplement: btaf082_Supplementary_Data [file btaf082_supplementary_data.pdf]

(Supplementary Materials) Tribus: Semi-automated discovery of cell identities and phenotypes from multiplexed imaging and proteomic data

Ziqi Kang, Angela Szabo, Teodora Farago, Fernando Perez-Villatoro, Ada Junquera, Saundarya Shah, Inga-Maria Launonen, Ella Anttila, Kevin Elias, Julia Casado, Anni Virtanen, Ulla-Maija Haltia, Anniina Färkkilä

1 Discussion on the overall approach and logic table design

Overall, Tribus is a hierarchical framework for cell-type assignment in multiplexed image datasets based on prior panel knowledge. Since Tribus is a "semi-automated" tool, in Figure S1 we defined the "human-in-the-loop" process in the Tribus workflow.

Regarding the input, running Tribus requires a marker expression table and a prior knowledge-based logic table. The marker expression table contains rows representing individual cells and columns corresponding to measured features, such as marker expression levels, cell eccentricity, or cell area. The original image is not directly involved in the cell type annotation step, but the user might need the image for later quality control. Please note that cell eccentricity or cell area does not necessarily need to be included. Those are cell morphology features and might be useful for identifying certain cell types (e.g. (1) a cell with eccentricity close to 0, which means roundness, is less likely to be a fibroblast, (2) tumor cells tend to have larger cell area). However, those features are more ambiguous for cell type annotation compared with typical marker expression. Thus, please be careful if you want to include those features.

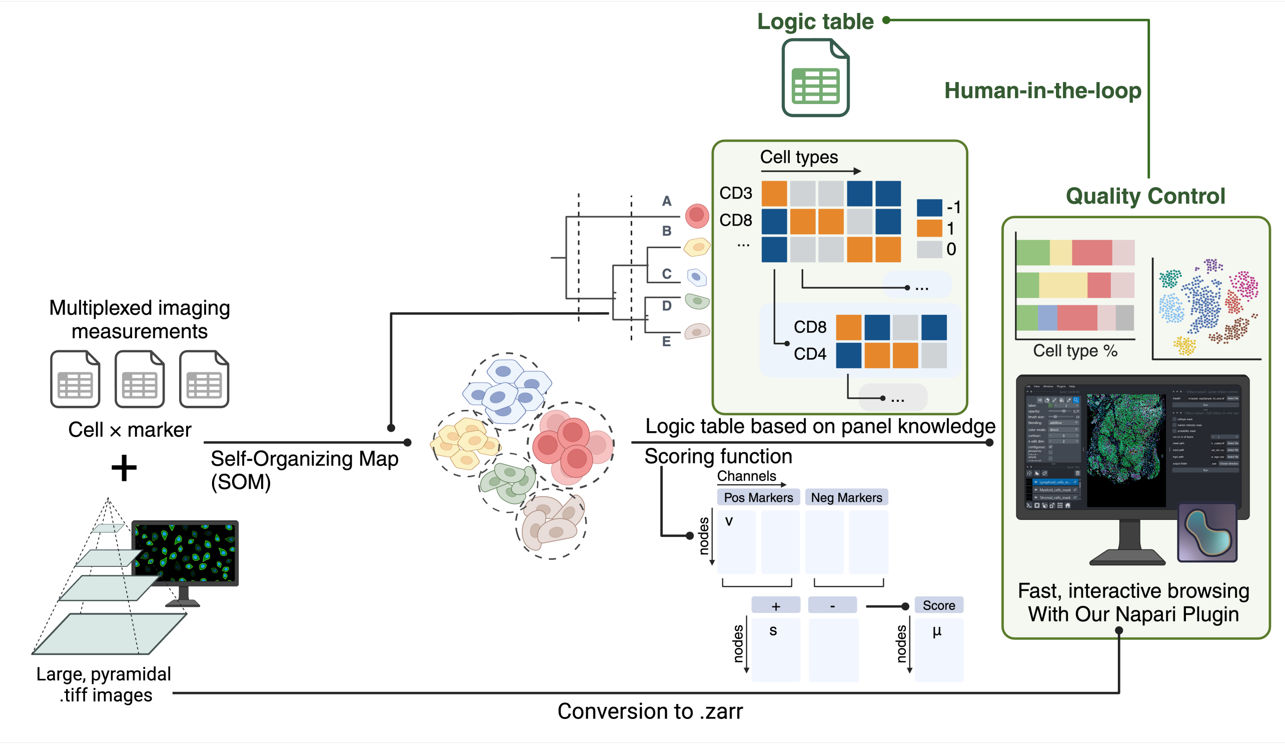

Figure S1: Tribus workflow, with green boxes to highlight the "human-in-the-loop" process in the Tribus workflow: (1) Logic table design and (2) quality control.

As mentioned in the main text, Tribus performance is highly related to the quality of the input dataset and the prior knowledge of expected cell types in the user-defined initial cell type logic table. Logic table design is also an important step before the automated processing. Here we will provide more notes on designing effective logic tables.

To improve the accuracy of cell type annotations, users can design a hierarchical logic table where major cell phenotypes with clear marker signals are separated first at higher levels. The following tables (Supplementary Table 1-3) compare one-level logic tables and multi-level logic tables. The example is based on the AML dataset, where the one-level logic table is from the original research of ACDC while the multi-level logic table shows a hierarchical design that refines cell type classification. To design multi-level logic tables: (1) We recommend including broad cell types at the global level, such as major immune or stromal cell populations, at the highest level of the hierarchy. These broad categories capture the primary phenotypes based on consistently robust marker signals. (2) Divide phenotypically different cell subtypes or rare cell types into sub-levels. We provide further examples to show how to build logic tables from known marker panels in real in-house experiments in Section 6.

Table 1: Logic table for AML dataset with 1 level

Marker	Basophils	CD4T	CD8T	CD16-NKs	CD16+NKs	HSPCs-1*	HSPCs-2*	HSCs*	MatureB	PlasmaB	PreB	ProB	Monocytes	pDCs
CD19	-1	-1	-1	-1	-1	-1	-1	-1	1	1	1	1	-1	-1
CD4	0	1	-1	0	0	0	0	0	0	0	0	0	0	0
CD8	0	-1	1	0	0	-1	-1	-1	-1	-1	-1	-1	-1	-1
CD34	-1	-1	-1	-1	-1	1	1	1	-1	-1	-1	1	-1	-1
CD20	-1	-1	-1	-1	0	-1	-1	-1	0	0	0	0	-1	-1
CD45	0	0	0	0	0	-1	-1	-1	0	0	0	-1	0	0
CD123	1	-1	-1	-1	-1	-1	1	-1	-1	-1	-1	-1	-1	1
CD11c	-1	-1	-1	-1	-1	-1	-1	-1	-1	-1	-1	-1	1	-1
CD7	-1	0	1	1	1	-1	-1	-1	-1	-1	-1	-1	-1	-1
CD16	-1	-1	-1	-1	1	-1	-1	-1	-1	-1	-1	-1	0	0
CD38	0	0	0	0	0	1	1	-1	0	1	1	1	0	0
CD3	-1	1	1	-1	-1	-1	-1	-1	-1	-1	-1	-1	-1	-1
HLA-DR	-1	-1	-1	-1	-1	0	0	0	0	-1	1	0	1	1
CD64	-1	-1	-1	-1	-1	-1	-1	-1	0	0	-1	-1	0	-1

Notes: In the table, HSPCs-1 represents CD34+CD38+CD123-HSPCs.
HSPCs-2 represents CD34+CD38+CD123+ HSPCs and HSCs represent CD34+CD38lo HSCs.

Table 2: Logic table for AML dataset with multiple levels

Marker	Basophils	CD4T	CD8T	NKs	HSPCs	B	Monocytes	pDCs
CD19	-1	-1	-1	-1	-1	1	-1	-1
CD4	0	1	-1	0	0	0	0	0
CD8	-1	-1	1	0	-1	-1	-1	-1
CD34	-1	-1	-1	-1	1	0	-1	-1
CD123	1	-1	-1	-1	0	-1	-1	1
CD11c	-1	-1	-1	-1	-1	-1	1	-1
CD7	-1	0	1	1	-1	-1	-1	-1
CD3	-1	1	1	-1	-1	-1	-1	-1
HLA-DR	-1	-1	-1	-1	0	0	1	1

Notes: This is the global level.

Table 3: Logic table for AML dataset with multiple levels

Marker	CD16-NKs	CD16+NKs
CD7	1	1
CD16	-1	1

Notes: In the second level, NKs are further divided.

Marker	HSPCs-1	HSPCs-2	CD34+CD38lo HSCs
CD34	1	1	1
CD123	-1	1	-1
CD38	1	1	-1

Notes: In the second level, HSPCs are further divided. HSPCs-1 means CD34+CD38+CD123-HSPCs. HSPCs-2 means CD34+CD38+CD123+ HSPCs.

Marker	Mature B cells	Plasma B cells	Pre B cells	Pro B cells
CD19	1	1	1	1
CD34	-1	-1	-1	1
CD38	0	1	1	1
HLA-DR	0	-1	1	0

Notes: In the second level, B cells are further divided.

For AML and BMMC datasets, logic tables were provided from the original research of ACDC and could be downloaded from link. Logic tables for Tribus were built based on the origin logic tables. For DCIS and HubMAP datasets, the logic tables for Tribus were built based on marker panels given in the origin studies. For the in-house datasets, please see details on how to build logic tables according to in-house experimental marker panels in Section 6.

All the logic tables used in the research can be found in the Synapse repository.

Finally, the outputs of Tribus include annotated cell types for each cell (Supplementary Table 4), which can be used for downstream analysis or visualization.

After receiving the output, users can perform easy quality control by (1) checking the marker expression heatmap by running the function heatmap_for_median_expression in Tribus package, or (2) if the original image is available, using the Napari plugin we provided in our GitHub repository. As we mentioned in the main manuscript, this Napari plugin enables users to run Tribus on one sample at a time, display results simultaneously, or load previously saved data. Quality control is performed by mapping cell-type labels on the original images.

If the cell annotation quality is not satisfying, it is usually due to: (1) some low-proportion cell types being dominated by some high-proportion cell types, (2) some markers are not performing as well as expected. The users may consider modifying the logic table by adjusting the hierarchical levels or removing low-quality markers.

Table 4: Example of Tribus output

CellID	Global	Sublevel 1	Sublevel 2	...	Final_label
1	Stromal			...	Stromal
2	Immune	B		...	B
3	Immune		Macrophage		Macrophage
4	Tumor			...	Tumor

2 Supplementary information on DCIS dataset

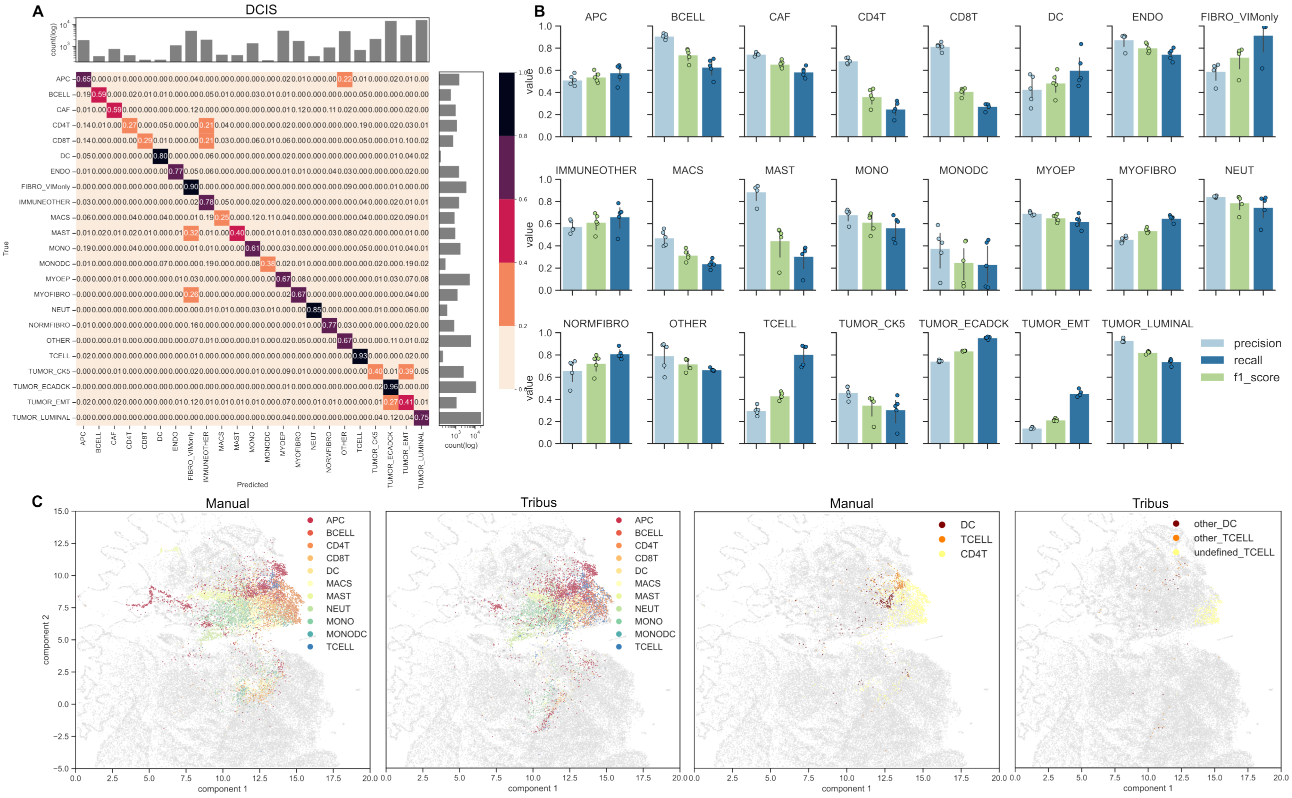

Figure S2: (A) Confusion matrix comparing Tribus labels and ground truth labels, with cell type number bar plots. The y-axis is log-scaled on the bar plots. (B) Per cell type metric calculated on DCIS Tribus labels, using three metrics (Precision, recall, and F1 score) for each. All analyses were repeated 5 times and the standard deviation for the error bar. (C) UMAP visualizations of manual and Tribus labels on the DCIS dataset, comparing the immune cell populations and the undefined immune subpopulations.

3 Explore on scoring function thresholds

Tribus cannot find cell types that do not exist in the input logic table, but it can return undefined cell types for further exploration with unsupervised clustering methods. Tribus has undefined_threshold and other_threshold. As we mentioned in the method part, if the maximum score of a cluster is smaller than a certain threshold set by users, the cluster will be labeled as the ”other” cell type. Similarly, if the difference between the maximum and second maximum score is smaller than a certain threshold, the cluster will be labeled as ”undefined”.

Here we provide instructions based on the comparison among different settings of thresholds. Tribus is robust to all the threshold settings in a certain stage, but if the threshold is set too big then it might crash the cell type call (Supplementary Figure 2). At the global level, Tribus can target the ”Other” cell population with a high accuracy of over 85%. At the second level, The accuracy remains from 60% to 70%.

Users can (1) keep those ambiguous cell populations as they are or (2) check the marker expression heatmap, if special expression patterns are identified, then update the logic table by adding another level and naming those cell populations.

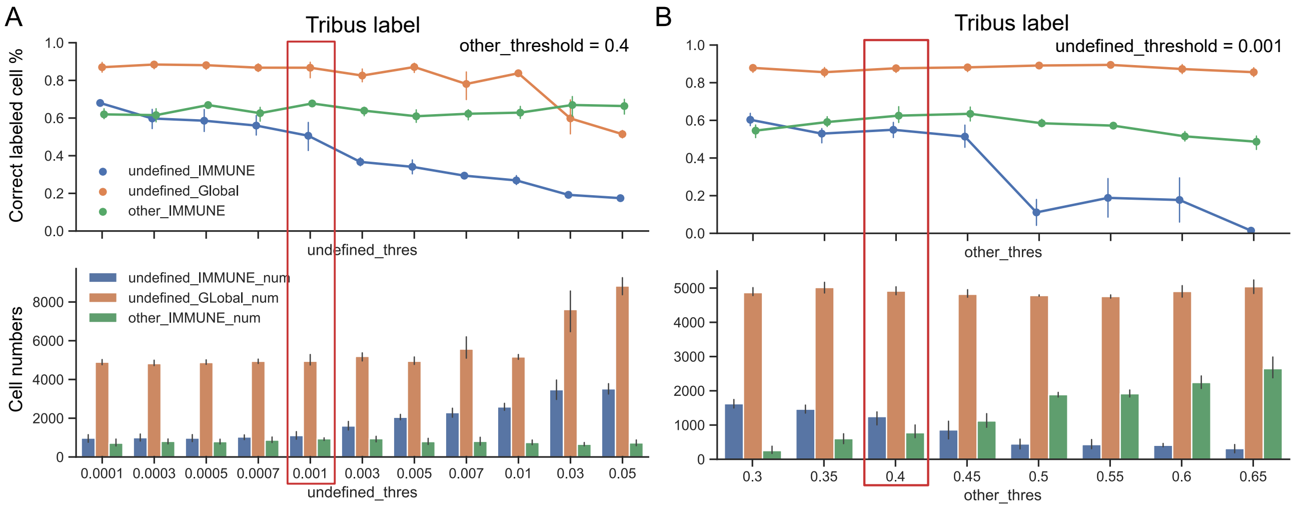

Figure S3: Compare different settings of thresholds. (A) With "other_threshold" = 0.4 (the setting used in the final analysis), change undefined_threshold. Calculate the correct labeled cell proportions and compare the number of cells in each group. Undefined_immune and other_IMMUNE correspond to IMMUNEOTHER in the ground truth label, and undefined_global corresponds to OTHER in the ground truth label. (B) With "undefined_threshold" = 0.001 (The setting used in the final analysis), change other_threshold. Calculate the correct labeled cell proportions and compare the number of cells in each group. All the analyses were repeated 5 times and the standard deviation for the error bar.

4 Discovery of novel cell populations from DCIS dataset

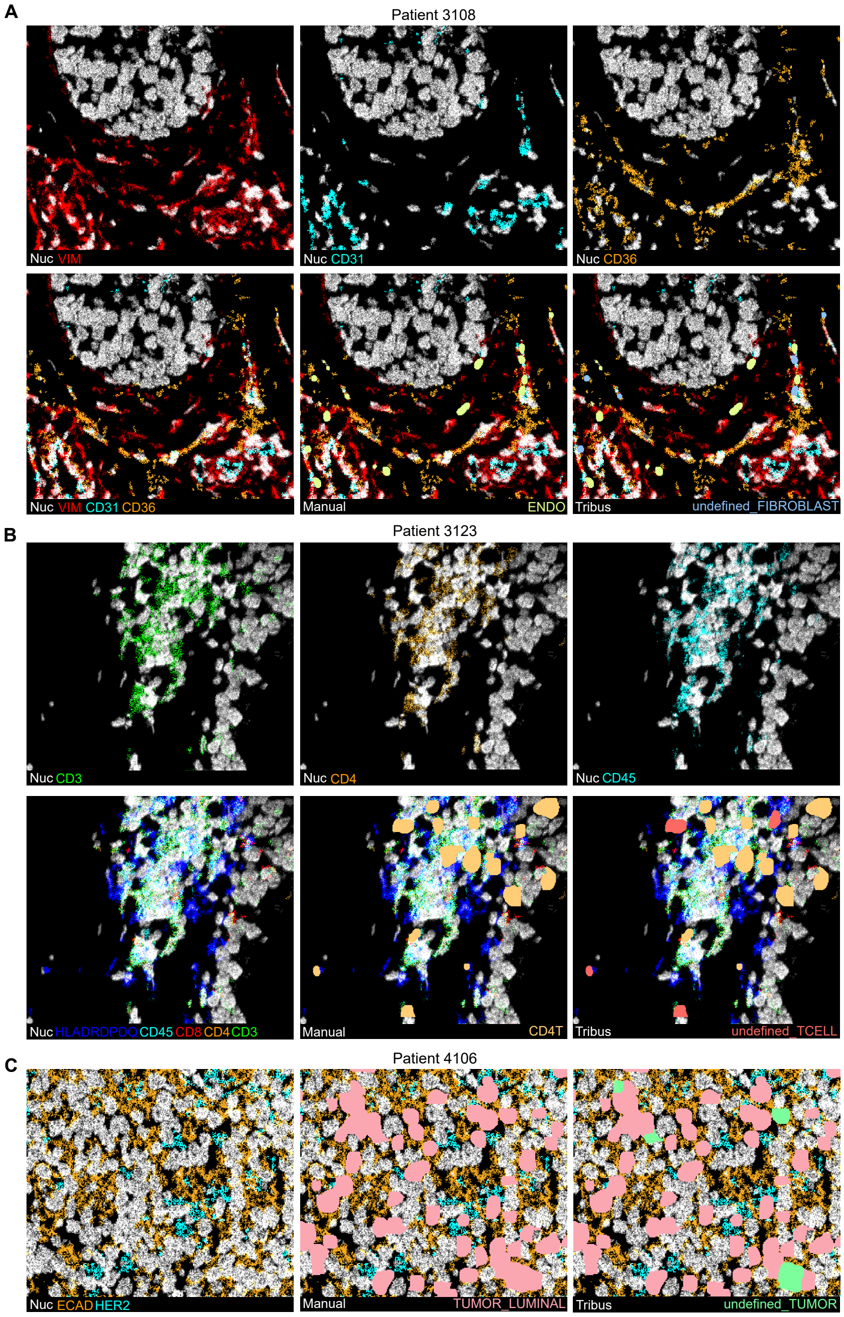

Figure S4: (A) Tribus identified CD36+CD31+ fibroblast subpopulation from the endothelial population. (B) The undefined T-cells have phenotypically higher marker expression compared with typical CD4T cells. (C) HER2- tumor luminal subpopulation.

5 Notes about benchmarking experiments

Table 5: Benchmarking metrics comparison (mean ± sd) per dataset

Method	Rand Index	Accuracy	F1 score	MCC	Kappa score
Tribus	0.8986 ± 0.0016	0.8743 ± 0.0044	0.8755 ± 0.0042	0.811 ± 0.0065	0.809 ± 0.0068
Astir	0.7547 ± 0.0101	0.3769 ± 0.0323	0.3875 ± 0.0354	0.3527 ± 0.0438	0.276 ± 0.0364
Scyan	0.8055 ± 0.0071	0.7315 ± 0.0137	0.7578 ± 0.015	0.6346 ± 0.0184	0.6211 ± 0.0208
ACDC	0.7601 ± 0.0332	0.6311 ± 0.0217	0.7045 ± 0.0312	0.5332 ± 0.0563	0.505 ± 0.0507

DCIS-lowplex dataset.

Method	Rand Index	Accuracy	F1 score	MCC	Kappa score
Tribus	0.9004 ± 0.0043	0.7157 ± 0.0163	0.7222 ± 0.0174	0.6726 ± 0.0193	0.6686 ± 0.0193
Astir	0.6401 ± 0.0009	0.1346 ± 0.0044	0.1282 ± 0.0049	0.1735 ± 0.006	0.1247 ± 0.0044
Scyan	0.8057 ± 0.0262	0.3343 ± 0.1033	0.3139 ± 0.1189	0.3346 ± 0.0823	0.2948 ± 0.092
ACDC	0.794 ± 0.0872	0.5092 ± 0.1596	0.5202 ± 0.1421	0.4647 ± 0.1379	0.4427 ± 0.1501

DCIS dataset.

Method	Rand Index	Accuracy	F1 score	MCC	Kappa score
Tribus	0.7947 ± 0.0363	0.7888 ± 0.0294	0.7908 ± 0.0328	0.7118 ± 0.0345	0.6999 ± 0.0434
Astir	0.5571 ± 0.0184	0.3711 ± 0.0333	0.4409 ± 0.0474	0.3561 ± 0.0296	0.2581 ± 0.0331
Scyan	0.7078 ± 0.1354	0.6042 ± 0.2008	0.5454 ± 0.2521	0.5045 ± 0.2211	0.4694 ± 0.2364
ACDC	0.2982 ± 0.0055	0.0 ± 0.0	0.0 ± 0.0	0.0 ± 0.0	0.0 ± 0.0

HubMAP-lowplex dataset.

Method	Rand Index	Accuracy	F1 score	MCC	Kappa score
Tribus	0.9003 ± 0.0145	0.5163 ± 0.0245	0.5609 ± 0.0232	0.4869 ± 0.0227	0.4807 ± 0.0233
Astir	0.828 ± 0.0302	0.1347 ± 0.0262	0.1797 ± 0.0312	0.1265 ± 0.0319	0.1136 ± 0.0289
Scyan	0.8977 ± 0.0035	0.4404 ± 0.0312	0.471 ± 0.0407	0.4106 ± 0.0275	0.4023 ± 0.0289
ACDC	0.3037 ± 0.0125	0.1546 ± 0.0126	0.1868 ± 0.0104	0.2256 ± 0.0086	0.1178 ± 0.006

HubMAP dataset.

Method	Rand Index	Accuracy	F1 score	MCC	Kappa score
Tribus	0.9537 ± 0.0254	0.8981 ± 0.0396	0.9016 ± 0.0417	0.8797 ± 0.0461	0.8764 ± 0.0484
Astir	0.9709 ± 0.0026	0.7627 ± 0.0221	0.7694 ± 0.0341	0.7336 ± 0.0238	0.7196 ± 0.0259
Scyan	0.9922 ± 0.0019	0.975 ± 0.0052	0.9748 ± 0.0074	0.97 ± 0.0061	0.9698 ± 0.0063
ACDC	0.9956 ± 0.0001	0.9832 ± 0.0003	0.9838 ± 0.0002	0.9796 ± 0.0003	0.9796 ± 0.0003

AML dataset.

Method	Rand Index	Accuracy	F1 score	MCC	Kappa score
Tribus	0.9719 ± 0.0019	0.8742 ± 0.0068	0.8868 ± 0.0065	0.8562 ± 0.0076	0.8552 ± 0.0078
Astir	0.5412 ± 0.026	0.1278 ± 0.0202	0.1234 ± 0.0128	0.1778 ± 0.0298	0.1136 ± 0.0177
Scyan	0.9916 ± 0.0033	0.9605 ± 0.0087	0.9611 ± 0.0076	0.9544 ± 0.0099	0.9543 ± 0.01
ACDC	0.9736 ± 0.0022	0.9288 ± 0.0051	0.9244 ± 0.0053	0.9179 ± 0.0058	0.9175 ± 0.0059

BMMC dataset.

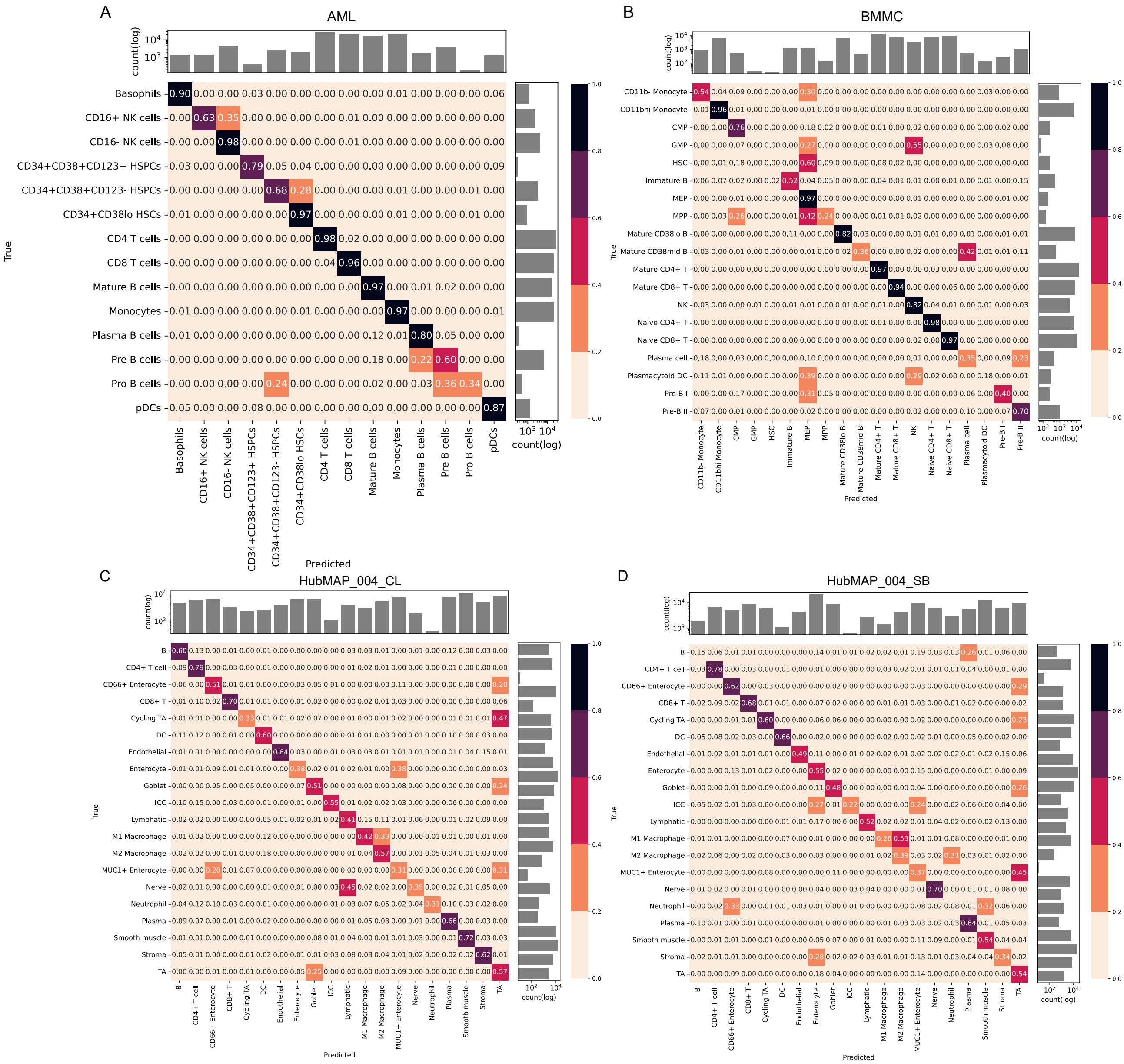

Figure S5: Confusion matrix comparing Tribus labels and ground truth labels, with cell type number bar plots. The y-axis is log-scaled on the bar plots. (A) AML dataset. (B) BMMC dataset. (C) HubMAP donor 004 colon dataset. (D) HubMAP donor 004 small bowel dataset.

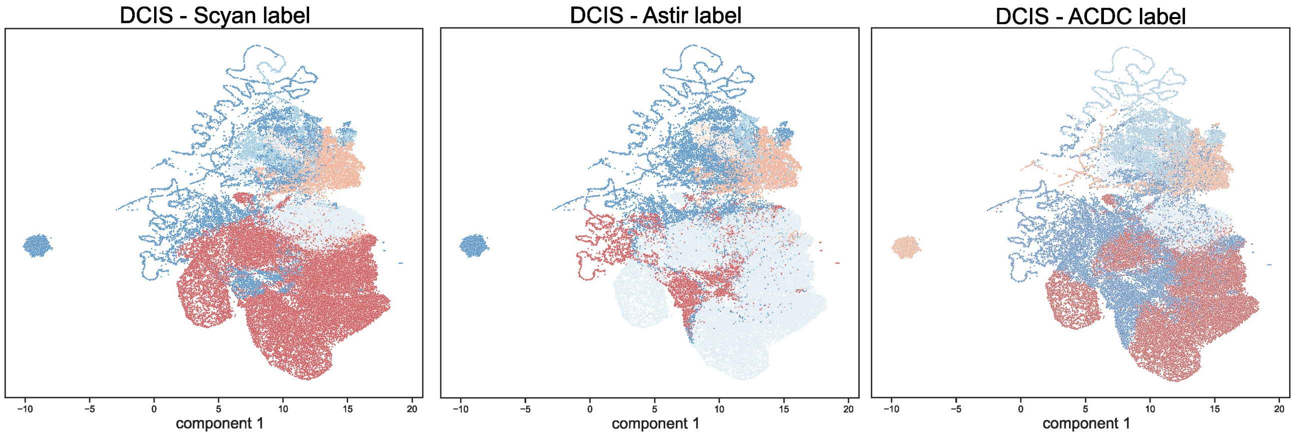

Figure S6: UMAPs representations of the annotations from all 4 methods on the DCIS dataset with low-plex cell types. The figures of the original label and Tribus label are in main Fig 2.

6 Notes about in-house datasets and logic table building

In the following Tables 5 and 6, we provide the panel information of Oncosys-Ova and NACT datasets. Note that the DNA 1-4 are all DAPI channels to indicate different cycles in the tCyCIF experiment. So in total, the panel includes 14 markers in the Oncosys-Ova dataset and 36 markers in the NACT dataset.

Table 6: Panel for NACT dataset

Marker	Purpose
Tuma	
Rabbit 488<br>Rat 555<br>Mouse 647	Background<br>Background<br>Background
Tuma	
CD11c<br>53BP1<br>CD1c	Dendritic cells<br>DNA damage<br>Dendritic cells
Tuma	
CD4<br>CD3d<br>CD20	T-cells<br>T-cells<br>B-cells
Tuma	
CD163<br>CD57<br>CD8a	M2 Macrophages<br>NK cells<br>T-cells
Tuma	
cCasp3<br>pSTAT1<br>yH2AX	Apoptosis<br>Interferon<br>DNA damage
Tuma	
CD15<br>Ki67<br>PD-L1	Mature neutrofils<br>Proliferation<br>ICP
Tuma	
IBA1<br>FOXP3<br>PD1	Macrophages<br>T-regs<br>ICP
Tuma	
E-cadherin<br>Vimentin<br>CD31	Tumor cells<br>Stroma<br>Stroma and blood vessels
Tuma	
P21<br>CK7<br>CD45	

In the NACT dataset, we dropped the CD20 panel in sample 05 due to unexpected staining patterns. We observed that the CD20 antibody also stained the nuclei of the cancer cells which resulted in abundant false positive B cells. Besides, the markers CD11c and CD1c for dendritic cells (DCs) are not working well according to the wet-lab specialists, thus we are not targeting DCs in the cell type annotation.

The panel was designed to identify the cell types (including cancer, stromal, and immune cells) in the tumor microenvironment. Some markers are functional (e.g. PD-L1, PD1) and thus are not included in the cell type annotation. With the following intuitions, we can build the logic table for the NACT dataset (Fig S5B).

- For the cancer cells, marker Ki67 was added to target proliferating and non-proliferating cancer cells - thus we should consider adding a sub-level to target proliferating and non-proliferating cells.
- For stromal cells, only 1 marker Vimentin was included and no further detailed cell types were targeted.
- Immune cells include Myeloids (Macrophages) and Lymphoids (CD8T cells, CD4T cells, B cells). Note that we can further divide macrophages into M1 (CD163-) and M2 (CD163+) subtypes since we have the corresponding marker.

Table 7: Panel for Oncosys-Ova dataset

Marker	Purpose
DNA1	Nuclear
CD4	CD4T cells
CK7	Tumor cells
HLA-A	
Vimentin	Stromal
DNA2	Nuclear
PAX8	Tumor cells
HLA-DPB1	
PD1	
DNA3	Nuclear
CD45RO	
CD3D	
CD45	
DNA4	Nuclear
IBA1	Myeloid
CD11c	Myeloid
CD8a	CD8T cells

The marker panel for the Oncosys-Ova dataset is quite simple (compared with the 36 markers in the NACT dataset), with many functional markers. According to the wet lab specialists, all markers worked properly. From the marker panel, we can see that the cell type markers target cancer, stromal, CD4 T, CD8 T, CD11c+ myeloid, and IBA1+ myeloid cells. Thus we can design a simple logic table with one global level (Fig S5A).

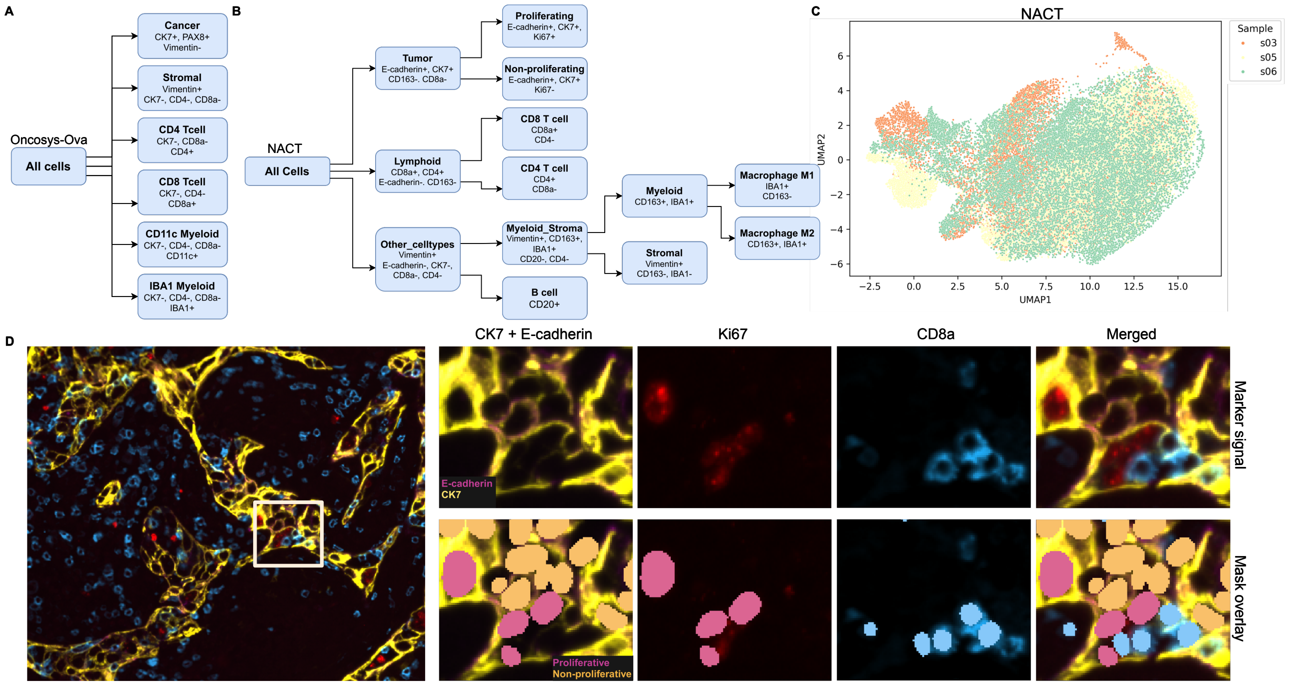

Figure S7: (A) Logic table for Oncosys-Ova dataset. (B) Logic table for NACT dataset. (C) UMAP projection of NACT dataset colored by sample origins. Cells are not clustered by origins, which illustrates a low batch effect. (D) Representative image of a tumor area, showing Tribus annotation of proliferation in tumor cells and tumor-infiltrating CD8+ cells.

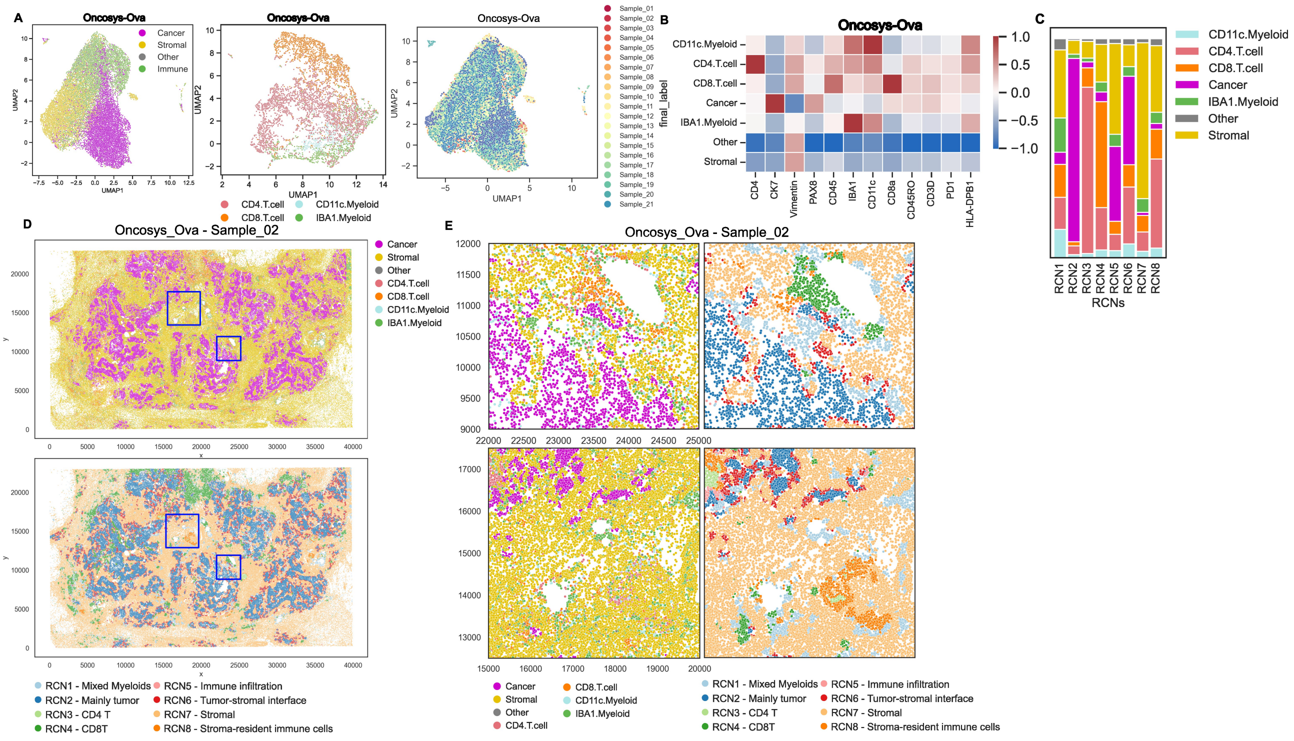

Figure S8: (A) UMAP projection of Oncosys-Ova dataset colored by the major cell types, immuno-subtypes, and the sample origins. Cells are not clustered by origins, which illustrates a low batch effect. (B) Heatmap showing the mean marker intensity of all cell types in the Oncosys-Ova dataset. (C) The stacked barplot shows the cellular proportion in each RCN. (D) Two representative images from Sample 02 show the tissue structures colored by cell types and the corresponding RCNs. (E) Scatterplots show a tumor-rich region (left) and a less-rich region (right), points are colored by cell type and RCNs.

7 Notes about Napari plugin usage

We provide notebook Tribus_napari_full_widget.ipynb in our repository for napari plugin. Run the chunks in the notebook and a napari window will pop out. You can check the quality of cell type calling and adjust the logic table correspondingly by directly running Tribus through the Napari interface or you can visualize previously assigned labels.

See detailed instructions in the README of the GitHub repository.
